# Public health education using social learning theory: a systematic scoping review

**DOI:** 10.1186/s12889-024-19333-9

**Published:** 2024-07-16

**Authors:** Ting Liu, Patrick Cheong-Iao Pang, Chi-Kin Lam

**Affiliations:** Faculty of Applied Sciences, Macao Polytechnic University, Macao, China

**Keywords:** Public health education, Social learning theory, Systematic scoping review

## Abstract

**Background:**

Public health education (PHE) in social environments plays a crucial role in mitigating the impact of public health events, especially with the recent surge in global incidents. Social learning theory (SLT) provides a strong theoretical foundation for implementing PHE. The objective of this study is to conduct a systematic scoping review of PHE using SLT, synthesizing the target populations, types of research, main findings, and future directions.

**Methods:**

The study followed the Preferred Reporting Items for Systematic Reviews and Meta-Analysis Extension for Scoping Review (PRISMA-ScR) guidelines. We conducted a comprehensive search of five electronic databases (Web of Science, Scopus, PubMed, ProQuest, and APA PsycInfo) for English articles related to PHE using SLT. Two reviewers independently screened the titles and abstracts. Descriptive statistics were utilized to analyze the characteristics of the articles included in the study, followed by a comprehensive narrative analysis of the results.

**Results:**

Research on PHE using SLT mainly focuses on adolescents, students, special patients, and vulnerable populations. The study sample includes seven research types and nine commonly used experimental methods. Four modes of PHE using SLT are identified, along with four types of summarized research results.

**Conclusion:**

PHE research based on SLT can be prioritized for preventing widespread infectious diseases, spreading fundamental public health information, and assisting patients with particular illnesses. To enhance the implementation of PHE, researchers and policymakers should integrate online and offline health education resources, ensure the accessibility of up-to-date information, and leverage digital technologies in PHE. More highly interactive and participatory health education courses will be established in social learning environments to encourage public participation in PHE.

## Background

Public health as the science and technology of improving the environment, preventing disease, prolonging life expectancy, promoting physical and mental health, and fostering the development of individual potential through organized social activities [1]. Therefore, the social aspect of public health has to be considered in practice. Public health is also based on the principles of social justice and equity which aims to address the underlying factors that affect public health, including policies and practices [2]. PHE is the outcome of the simultaneous growth of medical education and the advancement of modern medical careers, especially in the field of medical education. PHE is classified into four categories in the most recent academic research: promoting physical and mental health, developing healthy behaviors, enhancing environmental health, and preventing and controlling diseases [3]. In recent years, the emergence of global public health crises has had a significant negative impact on governments and the public. In light of the recent global COVID-19 pandemic, it has become clear that the general public lacks public health knowledge, which highlights the importance of strengthening PHE. In order to mitigate the significant impact and consequences of different public health events on the general public and society, it is crucial for governments to prioritize PHE. At the social level, the general public is the target audience for PHE. Through PHE, the government and relevant organizations can effectively raise public awareness of public health and enhance the public's ability to respond effectively to various public health hazards. Therefore, it is necessary to conduct PHE within the social environment.

SLT, which developed from behaviorism, is a social psychological theory that examines human learning in social environments. It was proposed by American psychologist Albert Bandura in 1952. It primarily focuses on the interaction between the learning environment, cognition, and behavior in influencing people's learning behavior, emphasizing the significance of the learning environment and individual behavioral factors [4]. The theory suggests that individuals can achieve effective learning outcomes by creating a conducive learning environment, engaging in continuous observation, and self-directed learning [5]. In recent years, most of the research related to SLT has focused on the fields of behavioristics, criminology, and psychology, with limited application in the field of public health. However, there are very few studies based on SLT, which may hinder the effective implementation of public health services. While a small number of public health studies utilize SLT, they primarily focus on higher education and professional talent development in public health, with limited emphasis on public and social PHE studies. SLT’s theoretical support for PHE is necessary for two reasons. Firstly, the public learning environment of PHE is situated in the social public space. Although the implementation of PHE in the public social space is more flexible and covers a wider range of education, the learning environment of a traditional classroom is more stable than that of PHE. Therefore, SLT is necessary to help address the challenges that PHE may encounter during the implementation of social public space. Secondly, traditional PHE is conducted under the supervision of schools and teachers, while public-facing PHE lacks supervision and relies more on individual learning behavior and self-learning efficiency. SLT can enhance learning efficiency by boosting learning motivation and personal learning behavior, thereby promoting the implementation effectiveness of PHE.

PHE is facing numerous challenges worldwide, with the most prominent ones being the dissemination of inaccurate health information [6], the rapidly evolving global health challenges, and the unequal distribution of health education resources. Among them, the gap and inequality in health education resources are worsening [7], particularly concerning the quantity and quality of educational resources [8]. Set against such a background, SLT provides the theoretical support to address these current challenges facing PHE. Our work aims to understand individual learning behavior in social learning environments for learning public health knowledge, which can help to overcome the current challenges in PHE.

Given the importance of using SLT in PHE, this study integrates the SLT perspective and seeks to investigate the impact of SLT-based learning environments and individual behavioral factors on PHE within social contexts. Additionally, this study aims to examine the distinguishing features of PHE in both online and offline settings.

## Methods

### Research question

To accomplish the research objective, the following research questions have been formulated:

a What are the target groups and research topics in this field of research?
b What types of research and data analysis methods were utilized in these studies?
c Which PHE models using SLT were used in these studies?
d What are their findings and results?
e What are the future trends and suggestions

### Search strategy

The reports in this systematic scoping review adhere to the Preferred Reporting Items for Systematic Reviews and Meta-Analyses Extension for Scoping Review (PRISMA-ScR) guidelines [9]. This review also adheres to Arksey and O'Malley's five-stage framework [10]. A three-phase search strategy appropriate for scoping studies is utilized. The initial phase involved a restricted search on PubMed and ProQuest. The search terms are repeatedly generated from the research subjects and related theoretical concepts. In the second phase, from our inception until October 16, 2023, we conducted searches using specific terms in the following five databases: Web of Science (WOS), Scopus, PubMed, ProQuest, and APA PsycInfo. The following strings are used to conduct a Boolean search across the four databases. WOS, PubMed, ProQuest and APA PsycInfo with the Booleans “AND”: All fields “public health education” AND “social learning theory”. Scopus with the Booleans “AND”: Title, Abstract, Keywords “public health education” AND “social learning theory”. Limited to articles published between January 2014 and September 2023. The third phase also involves manually searching the full text of all selected articles, as well as references from other similar review studies.

### Data selection and extraction

All retrieved records are exported to Zotero software, and any duplicate entries are removed. Two independent reviewers (TL and PP) initially screen the title and abstract of the article based on the inclusion criteria. Differences between two reviewers shall be resolved through negotiation with the third reviewer (CL). All standards are based on SLT, creating a social learning environment, and public-oriented health education. (1) This study is guided by SLT. (2) It focuses on educational behavior within the social environment. (3) The research is intended for the general public rather than professionals or students. Only articles, reviews, conference papers, early access, and proceeding papers are included. Detailed criteria for inclusion and exclusion are included in Table 1.

**Table 1:** Inclusion and exclusion criteria

Inclusion Criteria	Exclusion Criteria
Research that focuses on the general public rather than professionals or students in the health professions	Research that focuses on health professions students or practitioners
Research related to social learning theory or social environmental learning, and community-based	Research that has nothing to do with social learning theory, social environmental learning, or community
This directly relates to the study of public health education	Unrelated to research in public health education
Articles, Reviews, Conference Papers, Early Access and Proceeding Paper	Editorial Material, Book Chapters, Meeting Abstract, etc
Written in English	Written in other languages

### Data charting

We developed a data extraction form following the methodology guidance for scoping review provided by the Joanna Briggs Institute and made additional adjustments to the form after piloting five articles [11]. The items on the form included author, year, country, target groups, research topic, research and data analysis methods, model (a model for implementing PHE), findings, and outcomes. The data were graphed by two independent reviewers, and any sections that resulted in disagreement were resolved by consulting with a senior reviewer.

### Collating, summarizing and reporting the results

The data were collected, summarized, and analyzed, and descriptive statistics were used to characterize the sample articles. The findings were presented descriptively, accompanied by graphs and charts. The results were interpreted through a narrative synthesis to address the research questions targeted by the review. All the authors collectively confirmed the results.

## Results

**Fig. 1:**
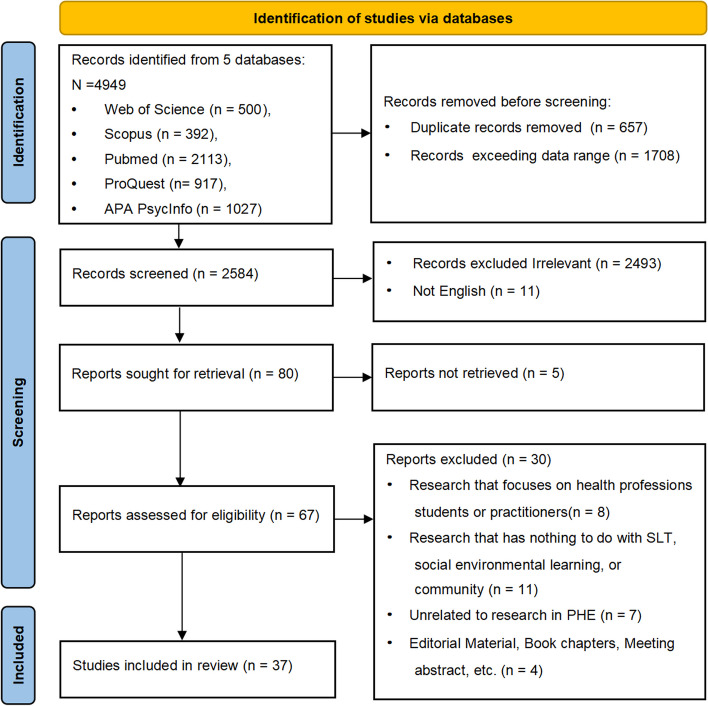
PRISMA flow diagram of selection process

As shown in Fig. 1, a total of 4949 articles were retrieved through the systematic search. After using the Zotero software to exclude duplicate articles, 2584 articles remained. After two reviewers independently screened the article titles and abstracts, 2493 articles that were not directly related to the research topic were excluded. Additionally, 11 articles that were not in English were also excluded. The remaining 80 articles were independently assessed in full by two reviewers, and 43 articles were excluded. Finally, 37 articles were identified for inclusion in the scoping review for this systematic review (see Table 2 [12–48]).

**Table 2:** Specific information about the articles included in the study

First Author, countries	Target groups	Research topics	Type of studies	Types of research and data analysis methods	Models	Findings and results
Alex Molassiotis et al., Hong Kong, China [12]	Vulnerable populations	COVID-19 prevention	Qualitative	Online in-depth qualitative interview	Academic–community platform, health education pamphlet	Design a human–computer interaction system and AI-driven Vaccine Communicator; Social distancing, promotion of vaccine uptake, eHealth education, preventive measures and early detection are the key to addressing COVID-19 reduction in the community
					Digital Health Literacy (DHL) interventions	
					Health communication campaign. Participatory social media strategies	
					Virtual reality game (VRG) educational programmer	
Tara Tancred et al., England [13]	Health education	Adolescent health	Standardized literature research	Intervention, triangulation	Full integration of health education into existing subject curricula	The elimination of boundaries between health, and academic education, teachers and students
					Integrate health and academic education	
					Enhance teacher-student relationship	
					Generalize learning from the classroom to the wider school environment	
					Provide training to parents	
					Providing training in resistance skills	
Aletha Y. Akers et al., USA [14]	Sexually-active women ages 14–21 years	Contraception and sex education	Quantitative and qualitative	Baseline questionnaire,	The Health Coaching for Contraceptive Continuation (HC3)	Health coaching is a new way for promoting contraceptive continuation in young women
				phone visits, exit interviews		
Laura Nyblade et al., USA [15]	Nursing students and ward attendants	HIV stigma	Intervention	Cluster randomized controlled trial, baseline assessment, post-intervention assessment	Participatory stigma-reduction training activities	Dristi interventions have the potential to change social norms and behavior of health workers
					Story boarding—script development and tablet content production	
					Pilot testing of tablet and in-person session materials	
Carlos Moreno-Leguizamon et al., England [16]	Black and minority ethnic people	Health services	Qualitative and case	Focus groups, formal/ informal interviews, electronic survey	Establish a learning alliance platform	Learning alliances and multicultural planning enhance comprehension of the connection between individual and group differences in health provision services
					Hold workshops	
					Create training packages	
Christina M. Karns et al., USA [17]	Parents of intellectual and developmental disabilities (IDD) children	IDD	Qualitative	Focus group discussions	Online knowledge and information base	Parents of IDD utilize the internet for accessing knowledge. Understanding the preferences and internet usage of these parents allows researchers and organizations to develop internet-based resources that cater to the specific requirements of these families
					Internet communities, forums, and blogs	
					Websites of hospitals, universities, academic journals, and other trusted institutions	
Emily Warren et al., England [18]	School staff and students	Bullying and adolescent mental health	Intervention	Randomized controlled trial, baseline surveys, interviews, focus groups	“Learning Together” interventions	“Learning Together” interventions were effective in reducing victims of bullying, reducing smoking, alcohol and drug use, and promoting mental health, mental functioning and health-related quality of life in students
					Building student commitment to the school community	
					Building healthy relationships by modelling and teaching pro-social skills	
Pamela Lamb, Canada [19]	Indigenous youth	HIV, sex education	Qualitative	Reflexive interviews	Participatory video	Participatory videos help address HIV/AIDS and sexuality education for indigenous youth in the community
Michelle Teti et al., USA [20]	College students	COVID-19 health education	Qualitative	In-depth interviews, sampling survey	Email, wall hang, ground stickers, handouts, visuals	An ecological model can be used to implement health education activities related to covid-19 on campus
Akshay Sood et al., USA [21]	Professional groups	Pneumoconiosis	Descriptive	Survey	Build virtual community practices—remote echo "clinics"	Tele ECHO may help deliver high-quality interdisciplinary care to miners in rural areas with high mortality rates in the USA
Charlotte Burman et al., Australia [22]	Disadvantaged and marginalized children	Integrated school-based health care needs of children	Qualitative and case	Semi-structured qualitative interviews	Free health services are arranged on school grounds	Service coordination, integration of education and health systems, trust, community partnerships, and perceptions of health are key factors that contribute to the success of school-based integrated health care models
					Streamline the support process with a centralized center, hosting and effective coordination of services	
					Integrating health and education systems	
					Build a trusting relationship with your family	
					Get support from community partners	
Victor Zogbochi et al., Benin [23]	Community's members	Patient with chronic diseases	Quantitative	Ant colony optimization algorithm	Online health communities	Increased socialization can be beneficial for patients
Patricia Solomon et al., Canada [24]	People living with HIV (PLHIV)	HIV rehabilitation	Quantitative and qualitative	Semi-structured interview, paper-based questionnaire	Experiential training program	Delivery of the community engaged program was viewed as feasible and acceptable; The ability for PLHIV to apply their advocacy knowledge and skills and access rehabilitation needs to be evaluated over time
					Learning manual	
					Delivering self-management programs to vulnerable populations such as PLHIV	
Mel Denehy et al., Australia [25]	Children under the age of 5 years	Child drowning prevention	Quantitative	Focus group interviews	Television campaign	Television campaigns can have a positive effect in preventing child drowning
Joanna Morrison et al., England [26]	Villagers in a Bangladeshi village	Diabetes	Intervention	Cluster randomized controlled trial, group interviews, focus group discussions, triangulation	Participatory learning and action (PLA) intervention	Community-based PLA intervention is an effective and cost-effective way to address diabetes in rural Bangladesh
					Community meeting	
					Organize community groups	
Nancy A. Shadick et al., USA [27]	Elementary school students in grades 2–5	Lyme disease (LD)	Intervention	Questionnaires, general linear regression analyses	School-based intervention based on elements of social learning theory and the Health Belief Model (HBM) in schools	short in-class LD education program and HBM can influence children's knowledge, attitudes, and self-reported preventive behaviors
					Short in-class educational program	
Melody Taba et al., Australia [28]	Adolescents aged 12 to 17	Digital health literacy	Quantitative and qualitative	Qualitative semi-structured interview, triangulation	Accessing online health information	Optimizing digital health literacy in young people is particularly important. Critical literacy capacity needs to be developed among adolescents through co-designed interventions
Sarah J. Javier et al., USA [29]	African American College Women	HIV prevention	Intervention	Questionnaires, follow up, linear regression analyses	Sister-to-Sister intervention	Single-session programs that combine experiential learning with educational methods may be more effective; Importance of culturally tailored interventions
					The CHAT intervention	
					Role-plays, discussion, and experiential learning activities	
					HIV/STD prevention video	
Katie Cueva et al., USA [30]	Community Health Aides/Practitioners (CHA/ p)	Cancer education	Quantitative	Evaluation survey	Online learning modules cancer basics	The online learning module addresses the lack of culturally relevant cancer education for CHA/Ps; Nearly all participants made health behavior changes to reduce their own cancer risk
					In-person cancer and wellness classes	
Jeanette M. Garcia et al., USA [31]	Young adults with autism spectrum disorder	Autism spectrum disorder (ASD)	Qualitative	Online survey, semi-structured interviews	Remote-based nutrition education sessions	The remote-based nutrition program met the benchmark of feasibility and gained wide acceptance among participants
Elena María Trujillo et al., Colombia [32]	Adolescents	Alcohol abuse	Qualitative	Participant observation, semi-structured interviews, focus group discussions	Adults set the example	The learning form of social learning theory plays an important role in the construction of adolescents' risk perception and behavior; There is a need to consciously assess how the role models set by adults and the ideas expressed in the media affect the attitudes and behaviors of adolescents
					Assessing the messages conveyed by the media	
Henry Quach, USA [33]	Elementary school age children	Oral health	Review	Randomized controlled trials	Lecture, printed material, demonstration, toothbrushing diary, game; video, Workshop, discussion	All the studies included in the analysis had interventions that were designed and led by adults, with children being involved solely during the implementation phase. It is suggested that future studies employ participatory research methods to gain a deeper understanding of the potential involvement of children in oral health education and research
Alfons Hollederer et al., Germany [34]	The unemployed	Mental health	Intervention	Randomized controlled trial; Computer-assisted telephone interviews, questionnaires, T-tests, Linear and (binary) logistic regression models	JOBS Program	The study will provide empirical evidence on the effectiveness of the JOBS Program on the reintegration and (mental) health of the unemployed in Germany
C. Vives-Casesr et al., Spain [35]	Boys and girls aged 13–17 years	Intimate partner, dating and gender violence	Intervention and quantitative	quasi-experimental educational intervention, on-line questionnaire	Lights4Violence: Training seminar with teachers	Lights4Violence is the first cross-national intervention study to promote positive relationships among adolescents
					Workshops with intervention groups	
					Short film exhibitions	
Mei-Fang Chen RN et al., Taiwan, China [36]	People with pre-diabetes	Pre-diabetes	Quantitative	Questionnaire survey	Diabetes shared care programs	When implementing mandated interventions, healthcare professionals should tailor their interactions with patients accordingly to increase knowledge, self-efficacy, social support, and healthy self-care behaviors in people with prediabetes
					Diabetes education classes	
Mastoreh-Sadat Ghoreishi et al., Iran [37]	Patients with type 2 diabetes	Self-care behaviors with type 2 diabetes	Intervention, descriptive, quantitative, and qualitative	Controlled intervention, questionnaires, Chi-square test, T-tests	Health education intervention	Health interventions based on social cognitive models have a positive impact on self-care of patients with diabetes
Beth A DeHoff et al., USA [38]	Parents of young children with special health care needs (CSHCN)	Needs of parents of CSHCN	Scoping review	Scoping review	Through digital media, particularly social media	Parents of adolescent children gain understanding and support and develop the ability to care for and advocate for their children through emotional and informational social support with each other; Online social support is most effective for young people of childbearing age, and social media and apps are most useful within the theoretical framework of social support
					Health application	
Nisha C. Gottfredson et al., USA [39]	Parents and adolescent	Adolescent smoking	Qualitative	Follow-up survey, generalized linear model	Increased antismoking communication	Parental involvement can provide protection for adolescents even when their parents smoke
Andrea Hickling et al., Canada [40]	Adolescents	Youth Concussion	Intervention	Cluster randomized trial, semi-structured Questionnaire, focus group interviews, survey, T-tests, multifactorial linear regression	Deliver a concussion awareness campaign and online showcase	Youth Concussion Awareness Network (You-CAN) will be an acceptable approach to advancing concussion knowledge sharing in Canadian high schools
					School-based, peer-led service-learning concussion intervention (Community service activities [38])	
					You-CAN web portal	
Olajide Williams and Ewelina M. Swierad, USA [41]	U.S. Blacks, stroke patients	Stroke health education model	Intervention	Randomized control trials, focus groups, semi-structured surveys	Hip Hop Stroke (HHS) (Musical, multimedia and interactive modules: Hip hop music, animated narrative cartoons, Video game, comic book, online portal)	Health education interventions for different communities need to consider three areas: arts, culture and science
					Design health education programming; Child mediated health communication model [40]	
Lindsey N. Horrell et al., USA [42]	Low-income, middle-aged participants	Chronic disease self-management	Quantitative	Chi-square tests	Chronic Disease Self-Management Education (CDSME) courses	CDSME courses offer a promising solution to curb the incidence of chronic diseases in middle age; Implementing CDSME marketing strategies can increase the participation of middle-aged people in low-income areas and can curb geographic and socioeconomic disparities in chronic disease management
D. J. M. A. Beaujean et al., Netherlands [43]	Primary school children aged 9–13	Lyme Borreliosis (LB) prevention	Intervention	Randomized control trials, short questionnaire, generalized linear mixed models	Online educational video game	Games are effective in improving knowledge and preventing behaviors related to ticks and tick bites. But the game did not outperform the flyer or control group on outcome measures. The educational video game can play a complementary role to other media within the health education program on ticks and LD aimed at children
					Leaflet	
Luke A. Vitagliano et al., USA [44]	College Students	Mental health and anxiety	Intervention	Control trials, reflective journaling, group conclusion	Nature-Based Mindfulness Training (NBMT)	NBMT integrates theoretical components from Mindfulness and ART as a psychoeducational group intervention that may benefit college students struggling with anxiety and improve mental health and well-being on college campuses
Yumei Li and Xiangbin Yan, China [45]	Users of online health community	Health Behavior	Quantitative study	Random-effects ordered logistic model	Online health community (OHC)	Online social relationships play an important role in promoting healthy behavior
Mohammad Afzal Mahmood et al., Australia [46]	Rural residents in areas with a high incidence of snakebites	Snakebite	Quantitative	Interview, structured questionnaire, Chi-squared test	Effective health education program and effective health system	Lack of preventive measures in areas with high incidence of snakebites. Communities in these areas need targeted health education programs. Health education campaigns through the mass media are a good start
					Community based health education	
					Mass media-based awareness campaigns	
Alison Caballero et al., USA [47]	Community members	COVID-19 vaccine	Quantitative	Online survey	Engaging community health workers as local trusted messengers	Guided by the theory of health behavior, tailored by the community, written in professional common language and delivered by trusted messengers, health education can have a positive effect on community members' understanding of COVID-19 vaccination knowledge
					Equip community leaders with a toolkit	
Eugene W. Farber et al., USA [48]	Patients of vulnerable populations	Spiritual and mental health primary care	Qualitative	Assessment	Patient-centered medical home (PCMH) model	The PCMH model can enhance cross-professional colleagues' understanding of the intersections of physical and behavioral aspects of health, administrative knowledge of institutional structures and policies, can promote inclusive and culturally responsive care environments, and scientific understanding of the application of quality monitoring and program evaluation efforts to meaningfully reduce health disparities and promote health equity

### Characteristics of studies

#### Countries of publications

**Fig. 2:**
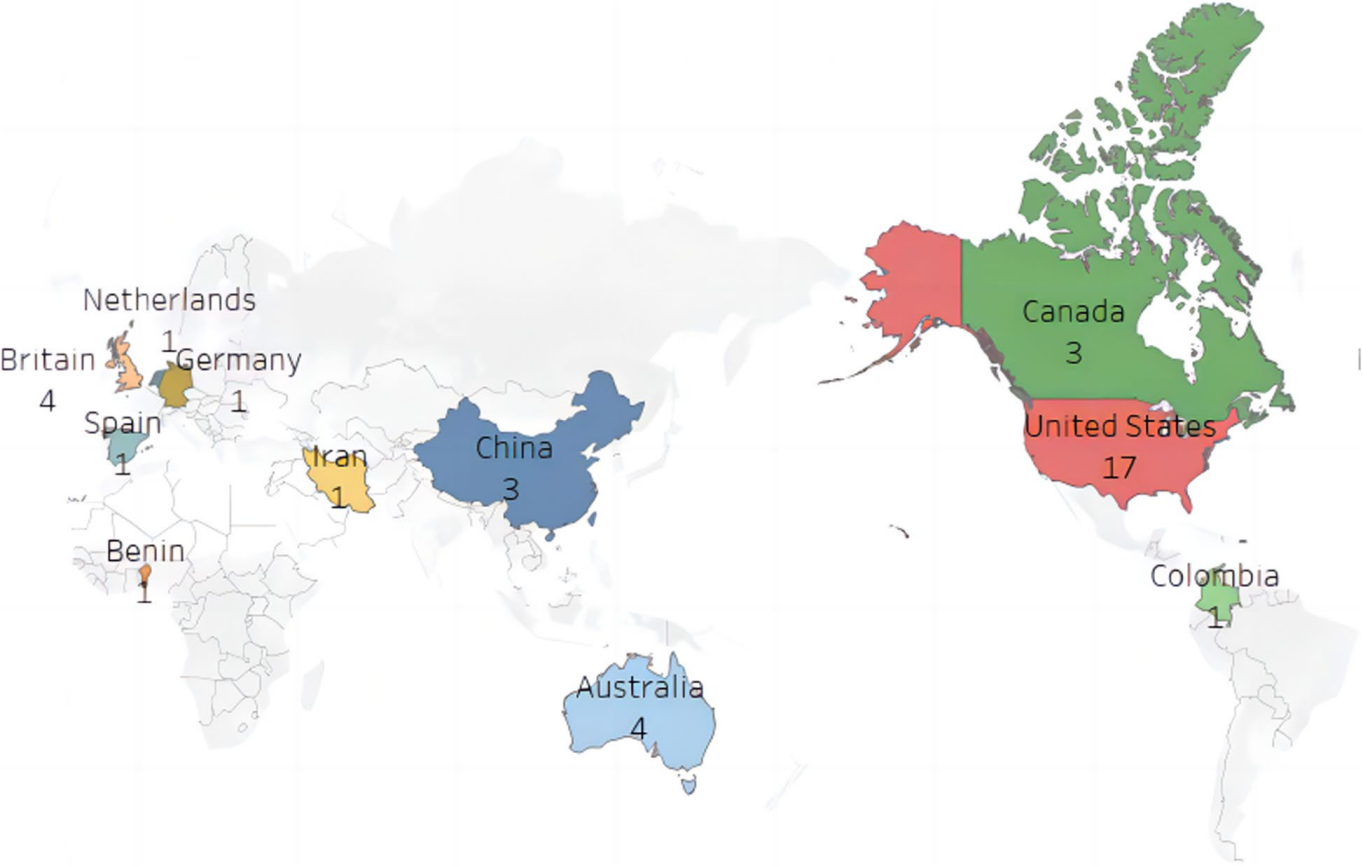
Distribution of 37 articles by country

Of the 37 studies, the United States, the United Kingdom, Australia, China, and Canada were the top five countries in terms of the number of papers, with the remaining studies mainly originating from six other countries. As shown in Fig. 2, the United States has the highest number of research projects among all countries, totaling 17. This indicates that the authors from the United States provide the most contributions to the research in SLT and PHE.

#### Target groups of the studies

**Fig. 3:**
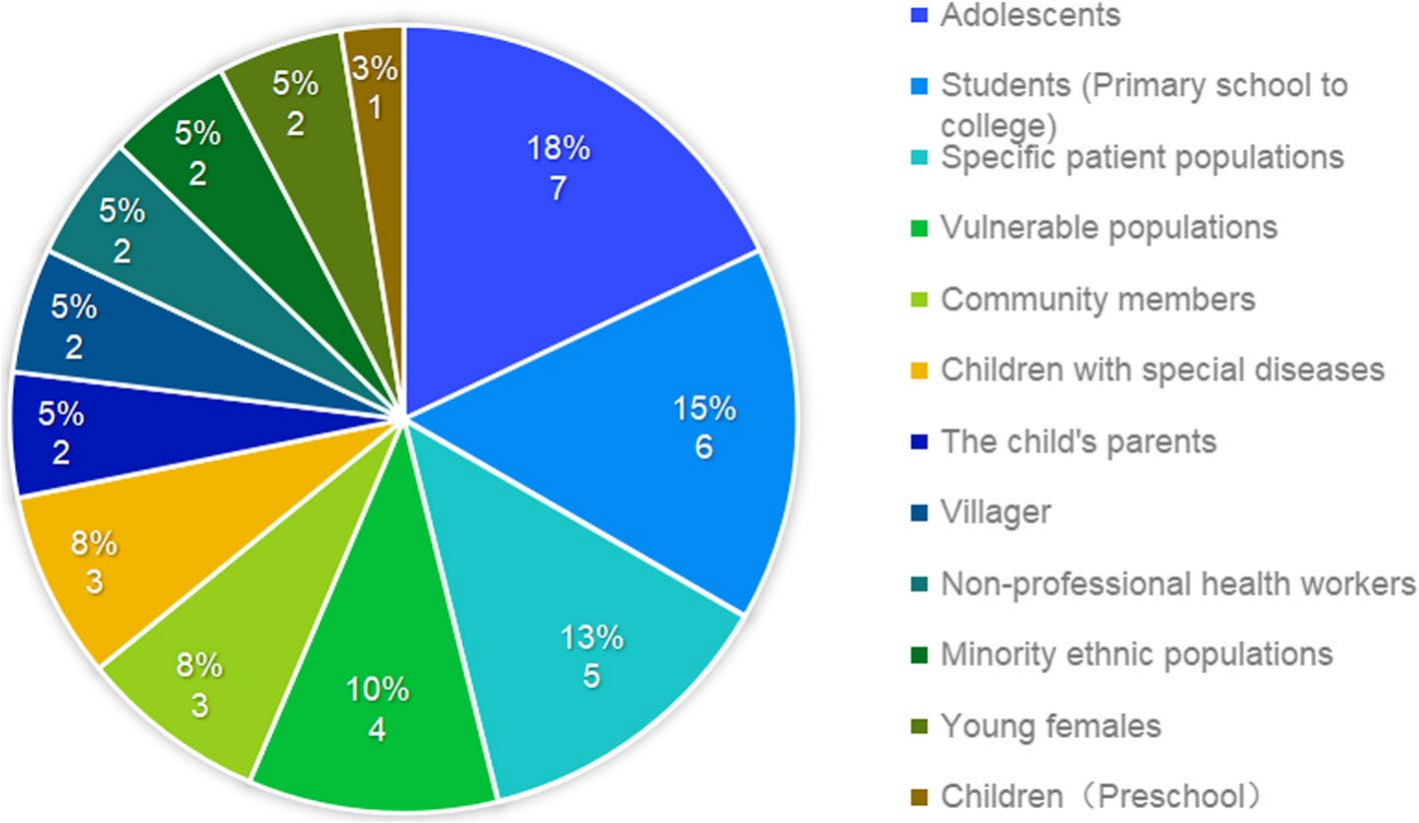
Types of target groups for studies

The studies covered 12 different target groups, including adolescents, students, vulnerable groups, community members, parents of children, young women, special patient groups, villagers, non-professional health workers, ethnic minorities, children in good health, and children with specific diseases. As shown in Fig. 3. Adolescents and students were the primary focuses of the research. The health of vulnerable groups and community members were also a concern. There have been relatively few studies on children, but a significant number of studies have focused on children with rare diseases and their parents. Relatively little research has been done on groups such as young females, special patients, ethnic minorities, rural areas, and non-professional health workers.

### Research topics

Research articles can be categorized into the following six themes: (1) sex education, encompassing HIV/AIDS prevention and treatment, as well as sex education and contraception; (2) special diseases, including diabetes, COVID-19, cancer, etc.; (3) rare diseases, such as Lyme disease in adolescents, mine pneumoconiosis, rural people snake bites, etc.; (4) non-disease health behavior education for adolescents, involving drinking, violence, smoking, etc.; (5) general public education, covering a wide range of health topics; (6) spiritual and mental health education research, such as adolescents' and college students' mental health, personal mental health, etc. The details of these topics are shown in Table 3.

**Table 3:** Research topic

Topic types	Diseases	Amount
Sex education	HIV and Sex education (Stigma, prevention, rehabilitation)	5
Special disease	Diabetes (Prevention, self-care)	7
	COVID-19 (Prevention, health education, vaccine)	
	Cancer	
Rare disease	Lyme disease	13
	Chronic disease	
	Snakebite	
	Pneumoconiosis	
	Intellectual and developmental disabilities (IDD)	
	Autism spectrum disorder (ASD)	
	Youth concussion	
	Stroke	
	Oral health	
	Drowning prevention	
	Needs of parents of young children with Special health care needs (CSHCN)	
Adolescent health behavior	Adolescent health problems (Violence, smoking, alcohol abuse)	4
Popular health education	Public health education, services, care	5
Spiritual and mental health	Spiritual and mental health	3

#### Types of studies

**Fig. 4:**
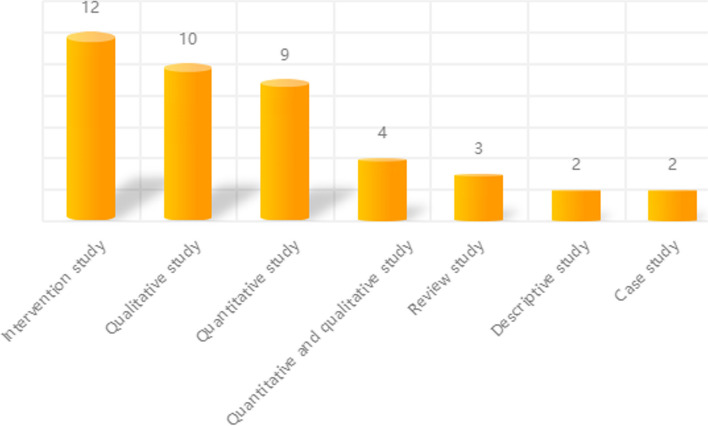
Types of studies

Sampling research is the most common method in intervention research, followed by quantitative and qualitative research. The four studies combined qualitative and quantitative methods. Three studies conducted sample reviews, while the other two focused on descriptive and case studies, respectively. In addition, two literature reviews and a systematic review are included. In contrast, the utilization of case studies and descriptive studies is more limited, as shown in Fig. 4.

#### Methods and intervention forms of studies

**Fig. 5:**
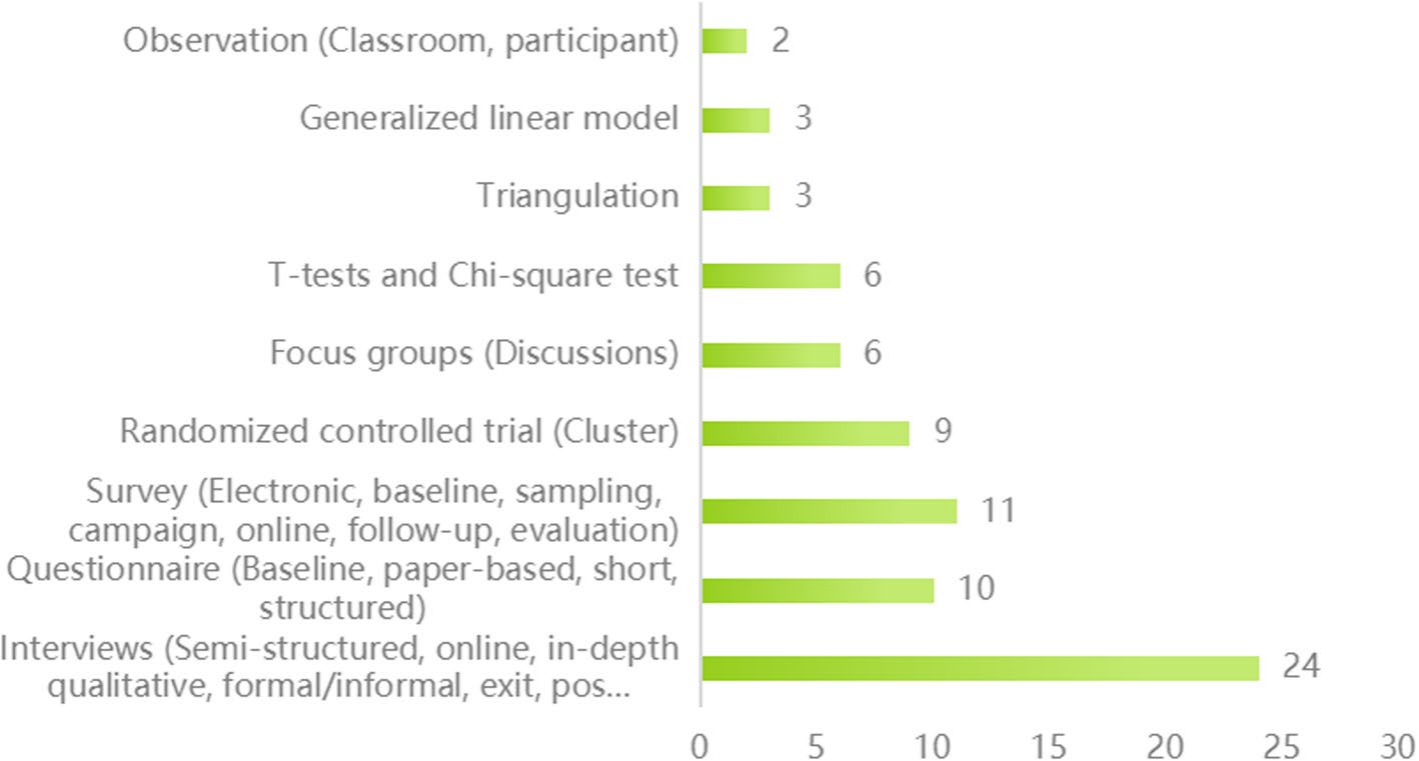
Types of research and data analysis methods

There are nine experimental methods commonly used in research, as shown in Fig. 5. Among these experimental methods, interviews, surveys, questionnaires, and (cluster) randomized controlled trials were widely used. Focus groups were used for qualitative studies, while T-tests and Chi-square tests were employed for quantitative studies. In addition, the methods of extended linear model, observation, and triangulation were also used.

In this study, we primarily identified four models for implementing PHE (Table 4). The first model involves offline intervention, followed by the production of materials related to PHE. Another type of intervention is online intervention. Finally, adults, serving as role models for healthy behavior among adolescents, evaluated and screened health education content posted on social media.

**Table 4:** Types of PHE models Using SLT

Types	Models	Amount
Offline interventions	In offline settings, public health intervention policies, strategies, and models are implemented by local governments, educational institutions, and other non-profit organizations. This includes related campaigns, workshops, courses, and conferences	22
Development of health education materials	Producing video games on public health; Developing training materials such as leaflets and information kits; Creating health education videos and films; Utilizing music, comics, and TV shows	13
Online interventions	Develop online education platforms, including websites, applications, communities, forums, and knowledge bases. In addition, PHE information can also be obtained through popular social media platforms such as Facebook, YouTube, Twitter, Instagram, TikTok, XiaoHongShu, and Sina Weibo	13
Model healthy behaviour to adolescents through adults	Adults model healthy behavior for adolescents and evaluate and screen health education content posted on social media	2

#### Definition of the effectiveness of PHE

Among the 37 studies, 18 defined the effectiveness of PHE through questionnaires, interviews, and scales after implementing health interventions like randomized controlled trials. They measured the effectiveness of interventions by establishing learning efficacy standards across various dimensions. Gottfredson et al. verified the effectiveness of family health education by tracking research subjects and measuring the positive interaction among adolescent gender, parental involvement time, and other variables 39. 5 studies defined the effectiveness of PHE based on participants' engagement in health education courses and acceptance of interventions. Garcia et al.'s study utilized interviews to assess the acceptability of intervention measures among participants, thus determining the effectiveness of PHE 31. 14 studies did not explicitly mention how to define the effectiveness of PHE. According to the analysis of the results of the effectiveness definition of the included study samples, it can be concluded that the criteria of the researchers' definition of the effectiveness of PHE are relatively unclear. There is no single measure or factor that can gauge the effectiveness of PHE. Defining effectiveness solely in terms of engagement and acceptance of PHE interventions and health courses is not comprehensive.

## Discussion

### Main findings and results of studies

First, offline education environments in PHE interventions led by governments, schools, communities, or other organizations can effectively enhance health education and promote the dissemination of PHE. Providing educational guidance or courses to the public or to specific patients and their families has a positive impact on their health. Implementing PHE in communities and schools can promote students' physical and mental health and reduce unhealthy behaviors.

On the other hand, online PHE has played an effective role in disseminating health knowledge, bridging social gaps, and promoting timely prevention. Online platforms, including websites, apps, communities, forums, and knowledge bases, effectively share PHE information. Social media and apps are effectively delivering health education messages.

Finally, adult health behaviors can influence adolescent health behaviors, making it crucial for adolescents to establish positive examples of healthy behaviors. Monitoring and screening health information on social platforms is essential to prevent minors from accessing inaccurate information. Therefore, the elimination of boundaries between academic education, schools, students, and classrooms has enhanced the effectiveness of public health communication in the school environment.

### PHE in a social learning environment

In the implementation of the PHE project, we may face various challenges, such as dealing with diverse individuals from different backgrounds 12. Factors contributing to intervention failure include changing views due to external circumstances. This makes it necessary to adapt interventions to evolving conditions and diverse educational goals. The target populations in PHE interventions are broad and diverse. To promote acceptance and comprehension of complex public health knowledge among diverse audiences, relying solely on traditional classrooms is insufficient. PHE should be implemented in a diverse social environment to attract a wider audience and provide comprehensive health education. This will help PHE reach a wider audience and achieve the best educational outcomes.

PHE should be conducted in a wider and inclusive social setting, with community public spaces being suitable venues. Community public spaces are commonly chosen by researchers for PHE implementation. These spaces allow people to engage in their daily activities and seek assistance conveniently from local community organizations. Communities share commonalities, including the natural and social environment, and may face similar public health problems. Therefore, implementing quick, easy, and flexible PHE measures in the community environment is a suitable solution to address the complexity of the target population during implementation.

Studies show that community-based public health engagement programs are feasible and effective 23. particularly in addressing regional diabetes issues 25. Community cohesion and a sense of belonging promote active participation in community activities. Successful implementation of PHE in community settings requires collaboration among community workers, government or non-governmental organizations, health workers, and community members. Community participatory PHE enhances health literacy and has been proven effective. The advantage of PHE in a social setting is the opportunity for full participation and interaction. Creating a conducive educational environment and supporting observational learning are crucial for PHE effectiveness. Participatory art education, such as participatory theater, has shown beneficial impacts on health education [49].

SDOH also have a significant effect on PHE. SDOH refers to the economic and social conditions that affect changes in the health status of individuals and groups [50]. The influence of individual behavior on social learning in SLT is closely related to the economic and social conditions that affect changes in individual and group health status in SDOH. Individual learning behavior is influenced not only by the learning environment, motivation, and other factors but also by personal income, education level, occupation, and other factors. Therefore, utilizing SDOH to examine PHE can assist us in accurately identifying the key factors influencing health and implementing appropriate health education and interventions. The distribution of social perception factors is often influenced by mainstream political ideology and public policies in the region [51]. The Special Health Status Report highlights the impact of social inequalities on health, including differences in factors such as income, education level, occupation, and living environment that contribute to inequitable health outcomes. Hollederer et al. mentioned that the unemployed are a vulnerable group in society, and the health status of the unemployed is often worse. However, reaching the unemployed with prevention and health promotion efforts is challenging 34. In PHE, the application of SDOH helps to promote equitable and inclusive health education, focusing on the health needs of vulnerable groups and reducing health inequalities. Parents of children with specific diseases belong to the category of vulnerable groups who have limited access to equitable health resources and require additional support from society, government, and policymakers 17. SDOH emphasizes multifaceted influences, including individual, societal, and system-level factors. Cross-sectoral and interdisciplinary partnerships are essential for the public health sector to effectively respond to health challenges. Lamb et al. applied an interdisciplinary participatory art approach to community PHE research with indigenous youth 19. Promote cooperation among all stakeholders from a health and wellness perspective to create a healthy environment and enhance public health. Based on PHE, comprehensive health intervention strategies can be developed. These strategies may include improving socio-economic conditions, promoting education and career development, enhancing the living environment, and strengthening community support. Such integrated intervention strategies can influence individual health behaviors and outcomes more comprehensively. Therefore, the application of SDOH in PHE contributes to a deeper understanding of the root causes and influencing factors of health problems. It can also contribute to the effective implementation of public health policies and interventions, ultimately achieving the goal of promoting overall social health and equity.

Observational learning based on SLT is significant in PHE, particularly in AIDS prevention education 28. Research supports the effectiveness of observational learning in health education. Adolescents’ health concepts and behaviors are strongly influenced by their external environment, with parents or caregivers having the greatest influence 30. Minors acquire public health knowledge through observational learning in social settings and may adopt unhealthy behaviors from adults. However, they may not have the ability to discern whether adults’ decisions are right or wrong. Studies show that even if parents smoke, it may not necessarily impact minors’ behavior. Nevertheless, adults play a vital role in educating minors about healthy behaviors and should serve as positive examples 38.

### Offline interventions for PHE with SLT

In this review, offline PHE interventions were commonly used. Offline interventions refer to PHE initiatives and behaviors carried out by local communities, schools, or organizations. These include the development of intervention policies, strategies, and models by the government, communities, schools, or relevant private organizations. Implementing appropriate health measures, educational models, or activities through offline modes can enhance the success and reach of PHE. Additionally, health intervention programs have been effective in addressing spiritual and mental health issues in specific populations 33. Offline interventions are also effective on campus, with integrated interventions successfully preventing youth violence in schools 20. Implementing offline interventions in school communities can reduce unhealthy habits and promote students' mental health, behavior control, and wellness. Furthermore, PHE interventions can also influence the behavior of health workers and target groups 14.

Offline implementation of PHE can also be achieved through organizing PHE activities, campaigns, workshops, courses, and conferences. These methods are commonly utilized in research. Through organizing various offline PHE activities, the enthusiasm of the public to participate in health education can be effectively enhanced, and the learning efficiency of health knowledge can be improved in an atmosphere of interactive learning. Studies have shown that when people participate in interactive learning, their ability to discover, imagine, and construct is improved, and their learning enthusiasm is enhanced [52].

The implementation of PHE guidance and curricula can have a positive impact on the general public, as well as on patients with specific diseases. Studies have found that short-term public health classroom learning programs based on SLT can effectively improve children's understanding of health knowledge and influence their health attitudes and behaviors 26. Other studies suggest that PHE in schools should aim to eliminate the divisions between health education and academic education, teachers and students, classrooms and schools, and schools and families 14. In other words, it is necessary to break the traditional classroom education mode and overcome the limitations of the traditional classroom in space, time, form, and content through more flexible and diversified education methods.

### Online interventions for PHE with SLT

Online PHE interventions are widely used and offer several advantages. They provide flexibility in terms of time and location, enabling individuals to access public health knowledge at their convenience. Online platforms have a broader reach compared to offline interventions, which are limited by the number of participants. Online PHE interventions can be continuous, allowing for updates and improvements in educational content. These interventions are particularly important for individuals facing mobility barriers and caregivers who struggle to attend offline activities. Research shows that online platforms serve as essential resources for health information and social support for people with specific diseases and their caregivers. Users rely on these platforms to gain knowledge, make informed decisions, and learn from the experiences of others 16. Online health education platforms have proven effective in supporting parents of children with special diseases and treatment needs 37 [53]. Optimizing digital health literacy among young people is critical due to their heavy use of the internet 27. Enhancing online platforms is necessary to cater to the educational needs of young people. Studies have also demonstrated the effectiveness of providing distance health education to young individuals 24.

Online platform researchers and designers gather user feedback to understand their needs and preferences, enabling them to create customized tools 30. Creating online virtual multidisciplinary communities of practice is an innovative approach to PHE, ensuring high-quality public health services for patients with specific diseases in specific areas 20. It is crucial for the online platform to fully meet user needs in order to retain users 22. Educational video games can complement other media in child health education programs, positively impacting children's health education 18. Videos and programs distributed through social media or other online platforms also contribute positively to PHE 18.

### Individual social learning behavior and PHE

According to the research, PHE interventions can be categorized into two forms: online and offline. These interventions have different impacts on individual learning behaviors in the social environment, which can be further classified into two main aspects:

PHE interventions can be categorized into online and offline forms. In the societal learning environment, an individual's learning behavior in PHE is influenced by two objective factors: the temporal factor and the spatial factor. Offline PHE requires individuals to go to a specific location at specific times, which limits their ability to participate in and complete their education. Offline interventions also have limited coverage and lack long-term continuity. On the other hand, online PHE provides flexibility and is not significantly impacted by timing and location restrictions. It requires more preparation, but it offers learners more control and convenience. Online platforms enable a focus on evolving social dynamics, as well as individual and group differences, in public health and service delivery.

In the social learning environment, the distinction between active and passive learning affects individual learning behaviors in PHE. Participants in PHE activities need to have a high level of learning initiative in order to successfully complete the educational process. Offline PHE requires individuals to actively seek out and participate in educational activities, contributing to their own learning initiative. Additionally, offline interventions organized by reputable institutions enhance participation and interactivity, thereby improving learners’ efficiency and motivation. Incorporating participatory visual arts activities in offline interventions for dementia patients has shown positive impacts on cognitive, social, and psychological functioning [54]. Jensen et al. study also supports the benefits of participating in artistic and cultural activities for sexual health [55]. On the other hand, online health education interventions do not require a high level of learning initiative, as individuals can access knowledge whenever needed. However, accessing health information online carries the risk of encountering incorrect information, which is a disadvantage of online PHE.

Online and offline forms of PHE have their own advantages and disadvantages, and integrating both forms can fully leverage their strengths. Some studies have integrated online learning modules with traditional face-to-face courses to educate learners on cancer and health. The results showed that participants who engaged in both online and offline learning were motivated to modify their health behaviors in order to reduce the risk of cancer 29. Whether PHE is conducted online or offline, both the intervener and education recipients need to have motivation and enthusiasm to promote the completion of educational activities. To enhance the effectiveness and engagement of PHE initiatives, researchers and government personnel should consider integrating multiple health education models. This approach will help achieve PHE goals and maximize its impact.

### Future trends in exploration

Future PHE research can focus on several areas, including prevention of large-scale infectious diseases, basic public health knowledge, health education for individuals with specific diseases and their families, as well as for vulnerable groups such as ethnic minorities and persons with disabilities. There is a lack of studies on the elderly population, despite the increasing global aging trend and the health challenges they face. Researchers should prioritize addressing the health concerns of the elderly and develop strategies for providing convenient and comprehensible PHE for this age group, particularly in chronic diseases, underlying conditions, and self-care. Adolescent health education and sex education remain important topics, but research may also shift towards including special patients, minority groups, and members of the broader community. The focus should be broadened to not overlook the health interests of minority populations. Additionally, PHE in the social environment should expand to include mental health, given the increasing mental and psychological pressures people face and their impact on personal and professional lives. Spiritual and mental health education should be emphasized in PHE.

In the future, the focus on social learning environments can be on enhancing public enthusiasm and participation in PHE by creating engaging and participatory educational atmospheres in community spaces. PHE interventions may combine online and offline approaches and collaborate with comprehensive intervention models to develop educational materials. Offline PHE can promote interaction and participation, increasing learners' motivation. Online PHE can enhance the dissemination of public health knowledge through user-friendly platforms and multimedia forms like AI interactive video games and electronic comics/animations. This combination of online and offline education, along with appropriate PHE materials, can greatly enhance understanding and facilitate widespread dissemination of PHE. The integrated use of these three forms can have a significant impact on PHE behavior in the social environment.

### Strengths and limitations

Introducing SLT into the field of PHE, the study explores the relationship between individual learning and behavior in social settings, focusing on the effectiveness of offline and online PHE. The study highlights the importance of removing boundaries in school settings and promoting cross-cutting PHE. This perspective on cross-disciplinary implementation offers a novel approach to enhancing the comprehensiveness and dissemination of PHE. It also presents a clear focus and direction for future PHE research based on SLT, which includes an emphasis on preventing large-scale infectious diseases, providing health education for patients with specific diseases, and addressing health issues among the elderly population. It is unique in the selection of research topics, methodological framework, and prospects of research conclusions, providing distinctive contributions and values for the research and practice of PHE. However, this review has several limitations. First, this review does not include non-English publications or gray literature from related fields. As a result, some pertinent studies may have been overlooked. Secondly, while each scope review is subject to our assessment, no individual quality assessment score is provided for each sample article. The reason for this is that such an assessment would not add value to the review.

## Conclusion

We have reviewed 37 studies of SLT based PHE. Most of them focus on adolescents and students, as well as sexual and disease-specific education, while it is fewer to study minorities and vulnerable groups, such young females and specific diseases. For PHE implementation, the combination of both online and offline PHE can be more effective by improving the real-time accessibility of health knowledge dissemination, and encouraging individuals to participate in PHE. The development of integrated PHE intervention models and diverse educational materials can promote the dissemination of public health knowledge and encourage broad participation. As suggested in our review, it is feasible and effective to offer participatory PHE in social learning settings, particularly within the community. The social learning environment should focus on fostering an interactive and participatory educational climate to increase public interest and participation in PHE. Additionally, researchers should prioritize the prevention of a wide range of infectious diseases, the dissemination of basic public health information, and health education for vulnerable populations, individuals with specific diseases, and their families. In conclusion, this work provides important references for further strengthening the implementation strategy and practice of PHE based on SLT.

## Data Availability

The author confirms that all data generated or analysed during this study are included in this published article. Furthermore, primary and secondary sources and data supporting the findings of this study were all publicly available at the time of submission.
